# Telomere Reprogramming and Cellular Metabolism: Is There a Link?

**DOI:** 10.3390/ijms251910500

**Published:** 2024-09-29

**Authors:** Maria P. Rubtsova, Denis A. Nikishin, Mikhail Y. Vyssokikh, Maria S. Koriagina, Andrey V. Vasiliev, Olga A. Dontsova

**Affiliations:** 1 Chemistry Department, Lomonosov Moscow State University, Moscow 119234, Russia; tais29998@mail.ru (M.S.K.); olga.a.dontsova@gmail.com (O.A.D.); 2 Shemyakin-Ovchinnikov Institute of Bioorganic Chemistry, Russian Academy of Sciences, Moscow 117437, Russia; 3 Department of Embryology, Faculty of Biology, Lomonosov Moscow State University, Moscow 119234, Russia; denisnikishin@gmail.com (D.A.N.); 113162@bk.ru (A.V.V.); 4 A.N.Belozersky Institute of Physico-Chemical Biology, Lomonosov Moscow State University, Moscow 119991, Russia; mikhail.vyssokikh@gmail.com; 5 Koltzov Institute of Developmental Biology, Russian Academy of Sciences, Moscow 119334, Russia; 6 Skolkovo Institute of Science and Technology, Center for Molecular and Cellular Biology, Moscow 121205, Russia

**Keywords:** telomere, development, alternative lengthening of telomeres, ALT, telomerase, metabolism, glycolysis, oxidative phosphorylation, OXPHOS

## Abstract

Telomeres—special DNA–protein structures at the ends of linear eukaryotic chromosomes—define the proliferation potential of cells. Extremely short telomeres promote a DNA damage response and cell death to eliminate cells that may have accumulated mutations after multiple divisions. However, telomere elongation is associated with the increased proliferative potential of specific cell types, such as stem and germ cells. This elongation can be permanent in these cells and is activated temporally during immune response activation and regeneration processes. The activation of telomere lengthening mechanisms is coupled with increased proliferation and the cells’ need for energy and building resources. To obtain the necessary nutrients, cells are capable of finely regulating energy production and consumption, switching between catabolic and anabolic processes. In this review, we focused on the interconnection between metabolism programs and telomere lengthening mechanisms during programmed activation of proliferation, such as in germ cell maturation, early embryonic development, neoplastic lesion growth, and immune response activation. It is generally accepted that telomere disturbance influences biological processes and promotes dysfunctionality. Here, we propose that metabolic conditions within proliferating cells should be involved in regulating telomere lengthening mechanisms, and telomere length may serve as a marker of defects in cellular functionality. We propose that it is possible to reprogram metabolism in order to regulate the telomere length and proliferative activity of cells, which may be important for the development of approaches to regeneration, immune response modulation, and cancer therapy. However, further investigations in this area are necessary to improve the understanding and manipulation of the molecular mechanisms involved in the regulation of proliferation, metabolism, and aging.

## 1. Introduction

Somatic cell proliferation depends on the availability of intracellular resources and is tightly coordinated with metabolism. To divide, a cell must accumulate proteins and lipids and replicate its DNA to produce a daughter cell. Eukaryotic cells cannot divide endlessly; the end-replication problem restricts the cellular proliferation capacity [1,2]. Telomeres—repetitive sequences located at the ends of linear chromosomes—provide the defense of genetic information from loss during the replication process and discrimination from internal double-stranded breaks. However, telomeres shorten with each cellular division, and once they reach a critical length, cells induce senescence [3,4]. This mechanism likely helps a multicellular organism eliminate cells that may have accumulated mutations due to multiple rounds of replication. Telomeres shortening limits the number of times eukaryotic cells can divide. This is usually sufficient for most somatic cells, which do not divide extensively; however, it is not sufficient for special cells like stem and germ cells, which must divide constantly [5,6]. Tissue regeneration, immune response, and tumor formation—all of which highly depend on proliferation and differentiation—require increased cell growth. Indeed, this growth is supported by telomere lengthening and the activation of anabolism—a pathway that uses energy for biosynthetic processes [7,8,9].

Telomerase—a specialized reverse transcriptase—is activated to elongate telomeres in vertebrates under conditions that support increased proliferation [6,10,11]. The alternative lengthening of telomeres (ALT) through break-induced replication of telomeric DNA supports telomere lengthening in some stages of embryonic development and in telomerase-negative cancer cells [12]. This review aims to provide an overview and discussion of the mechanisms underlying telomere elongation during gametogenesis, early development, and immune response, as well as any potential connections to cellular metabolism programs.

## 2. Telomeres: Structure and Regulation

### 2.1. Telomere Structure

**Figure 1:**
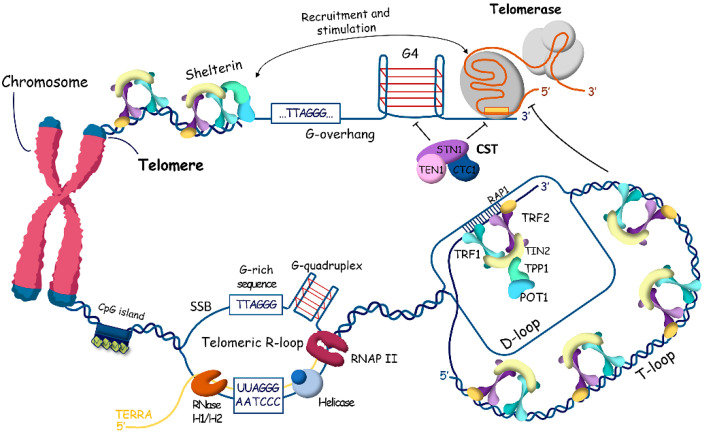
Human telomeric chromatin structure. The long double-stranded region of telomeric DNA is packed with nucleosomes in specific columnar arrangement and the very ends of telomeres are organized by the shelterin complex composed of telomeric repeat binding proteins 1 (TRF1) and 2 (TRF2), TRF1-interacting nuclear factor 2 (TIN2), TIN2-interacting protein (TPP1), protection of telomeres protein 1 (POT1), and repressor/activator site-binding protein 1 (RAP1). To prevent the degradation and reparation of chromosome ends, T-loops and D-loops protect telomeres. G-rich 3′-overhangs form G-quadruplex structures that stimulate telomerase to synthesize telomeric repeats and prevent the synthesis of the C-rich strand by the CTC1-STN1-TEN1 (CST) complex. Transcription of subtelomeric regions produces TERRA (TElomere Repeat containing RNA), which promotes R-loop formation stabilized by G-quadruplex structures at the opposite strand.

The very ends of linear eukaryotic chromosomes are organized in special DNA–protein structures called telomeres. Telomeres protect linear eukaryotic chromosomes from the loss of genetic information during replication and false recognition as DNA breakage sites. Vertebrate and some invertebrate telomeric DNA are composed of long (several kilobases) double-stranded regions of TTAGGG (Figure 1) repeats, flanked by a 3′-end single-stranded G-overhang [13,14,15]. The telomeres shorten with every cell division, serving as a molecular clock that limits the proliferative lifespan of cells. Critically short telomeres activate mechanisms of cellular senescence, promoting cell death [16,17]. Telomeres may be elongated by telomerase [18] or through an alternative mechanism based on homologous recombination [19]. The initial telomere length of somatic cells is derived from germline cells [20]. Stem cells need to preserve telomere length so they can regenerate effectively. Cancer cells also use mechanisms to maintain telomere length, allowing them to keep dividing for longer periods. Telomere length is important for the lifespan of cells and organisms. If it is not properly regulated, it can lead to issues with regeneration, premature aging, tumor formation, and growth with subsequent cancer progression.

The shelterin complex associates with mammalian telomeres to regulate various aspects of telomere function and contains six protein subunits: telomeric repeat binding proteins 1 and 2 (TRF1 and TRF2), TRF1-interacting nuclear factor 2 (TIN2), protection of telomeres protein 1 (POT1), POT1 and TIN2-interacting protein (TPP1), and repressor/activator site-binding protein (RAP1) [21,22,23].

**Figure 2:**
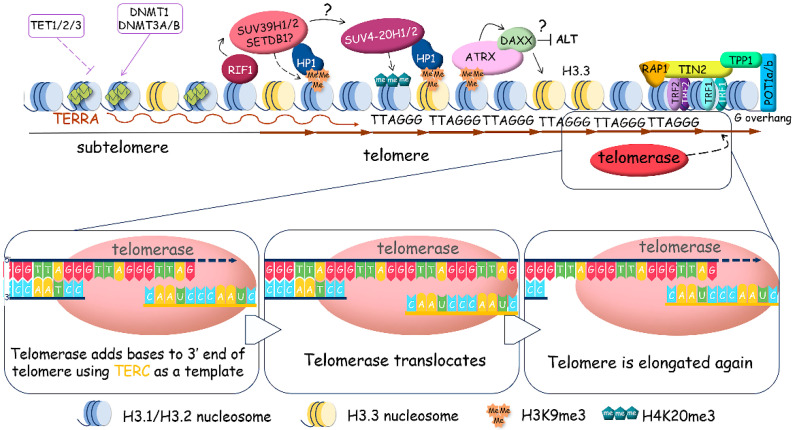
Scheme of telomerase action at telomeres. Telomeric chromatin is organized in a closed state. Subtelomere DNA is methylated at CpG dinucleotides. Histones are characterized by heterochromatin modifications (H3K9me3 and H4K20me3), which are established by SUV39H1/2 and SUV4-20H1/2. Despite the heterochromatin status of telomeric regions, subtelomeric regions contain promoters that provide low-level transcription of the long non-coding RNA TERRA. The a-thalassemia/mental retardation syndrome and X-linked (ATRX) and death-domain-associated protein (DAXX) complex (ATRX/DAXX complex) stimulates the accumulation of H3.3 nucleosomes and prevents the homological recombination of telomeric regions. The shelterin complex, through TPP1, loads telomerase to the very end of the telomere and stimulates the synthesis of telomeric repeats in a processive manner, allowing the enzyme to translocate along the telomere and add more telomeric repeats after the synthesis of a single repeat.

**Figure 3:**
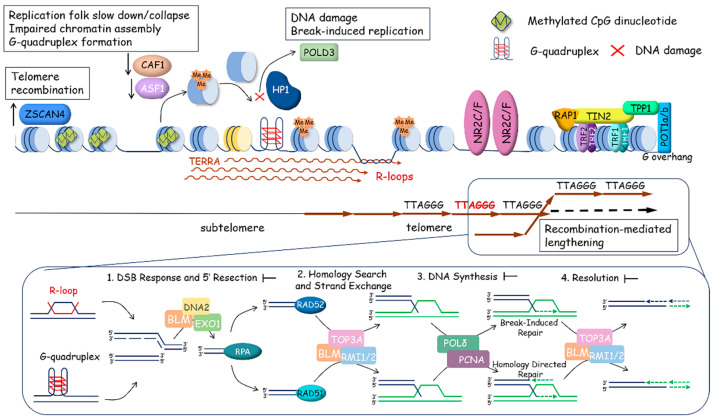
ALTernative mechanism of telomere maintenance. Absence of ATRX at telomeres promotes recombination at telomeric chromatin. Repressive marks are reduced, and transcription of TERRA is stimulated, leading to putative recombinogenic DNA–RNA hybrids (R-loops). R-loops stimulate the formation of G-quadruplexes, leading to replication fork stalling followed by DNA damage response, activation of homology searching, and strand exchange, assisted by RAD51 and RAD52 accessory factors. After DNA synthesis, break-induced and/or homology-directed repair finalize the process of recombination. The accumulation of variant repeats in ALT cells resulting from telomere recombination can induce the binding of NR2C/F transcription factors to telomeres, leading to chromosomal rearrangements and genomic instability.

Shelterin caps the ends of chromosomes and organizes the T-loop structures [24], but the long double-stranded region of telomeric DNA is packed with nucleosomes in specific columnar arrangement. Telomeric nucleosomes are less stable and more dynamic structures with unwrapping of DNA ends compared to canonical nucleosomes [25,26,27,28]. Human telomeric chromatin is enriched in histone H3 trimethylated at Lys9 (H3K9me3), a marker of constitutive heterochromatin, which coexists in telomeres with H3K27me3, a marker of facultative heterochromatin. Taken together, telomeric structure can be viewed at two different scales: the higher-order chromatin architecture, which regulates telomere accessibility, and the more compacted structure of telomeric ends, which influences specific telomere replication mechanisms (Figure 1, Figure 2 and Figure 3).

In addition to T-loops, telomeric DNA is involved in the formation of two other noncanonical structures: the G-rich chain forms G-quadruplexes [29,30], and the transcription of telomeric regions stimulates the formation of R-loops, which are RNA–DNA hybrids [31,32] (Figure 1). For a long time, the telomeric region of chromosomes was considered transcriptionally silent. However, recently, transcription resulting in the synthesis of long non-coding RNA named TElomere Repeat containing RNA (TERRA) was reported [33,34]. RNA polymerase II starts from promoters in the subtelomeric regions and synthesizes G-rich heterogeneous in length (from 100 nts to more than 9000 nts in mammals) and consists of RNA 5′-UUAGGG-3′ repeats in the direction of the end of chromosomes. An increased level of TERRA correlates with the accumulation of R-loops at telomeres, and the study demonstrated that the number of R-loops is higher at critically short telomeres [35]. Indeed, TERRA R-loops are stabilized at critically short telomeres, interfering with replication, which causes replication stress and the induction of double-stranded breaks [36,37]. The activated DNA damage response promotes homology-directed repair to elongate telomeres.

In the majority of vertebrate cells, telomeres shorten with each division cycle because of the end-replication problem and nuclease action. However, telomeres must be maintained in cells with increased proliferation potential, such as germ, stem, and cancer cells. At the beginning of embryonic development, telomeres elongate very efficiently to obtain the length necessary for many divisions before birth and throughout the life of the organism. There are two basic mechanisms of elongation of telomeres known to be used in cells: elongation of the 3′-overhang by telomerase, followed by filling in the C-rich strand by DNA polymerase a; and the alternative lengthening of telomeres (ALT), which is based on homology recombination.

### 2.2. Telomere Lengthening by Telomerase

Telomerase reverse transcriptase elongates the 3′-overhang of the telomeric DNA copying template region of the telomerase RNA component (TR) [10,11]. The telomeric 3′-overhang is used for primer base pairing, with 3′-end binding to the template region of the TR, which initiates the telomerase catalytic cycle (Figure 1 and Figure 2).

The interaction with telomeres is the first and main step of the catalytic cycle of telomerase. The dynamic structures of telomeres and shelterin play an important role in regulating telomere accessibility for telomerase. Shelterin can protect telomeres by stimulating the formation of T-loops and/or end-capping the telomeric 3′-overhang [21]. Alternatively, it can recruit telomerase to telomeres and stimulate telomerase processivity in the addition of multiple telomeric repeats [38,39,40]. The TEL-patch (TPP1 glutamate (E) and leucine (L)-rich patch) of the OB-fold (oligonucleotide/oligosaccharide-binding) of TPP1 directly interact with the TEN (telomerase essential N-terminal) domain of TERT during the S-phase of the cell cycle after genome replication. The process of this interaction is highly dynamic, and multiple tentative interactions occur before contact stabilizs through base pairing of the telomeric end with the template region of telomerase RNA [41,42]. TPP1 should help telomerase designate the telomeric tail and exclude binding with internal regions of telomeres.

Interestingly, POT1 has a controversial role regulating telomerase attraction. When bound to the internal regions of the ssDNA overhang, it stimulates telomerase association and activity; however, its interaction of the POT1 with the very end of the telomere blocks telomerase binding and inhibits telomere lengthening [43,44]. Moreover, POT1, in coordination with TERRA and hnRNPA1 [45], removes replication protein A (RPA) from telomeric ssDNA, which inhibits telomerase activity [46]. POT1 binding is necessary to protect telomeric ssDNA [47,48], and its association with TPP1 stimulates telomerase processivity [49].

### 2.3. ALTernative Mechanism of Telomere Lengthening

In some cells where telomerase is repressed, the DNA repair pathway, particularly homologous recombination (HR), is activated to maintain telomere length through the ALT mechanism (Figure 3) [19]. The ALT pathway is associated with the inactivation of a-thalassemia/mental retardation syndrome and X-linked (ATRX) and death-domain-associated proteins (DAXX) [50]. ATRX and DAXX are the components of a multifunctional chromatin-remodeling histone chaperone complex responsible for replication-independent localization of histone H3.3 at telomeres and pericentromeric chromatin [51,52,53]. ATRX interacts with DNA methyltransferase 1 (DNMT1). Mutations of lysine 27 (K27M) in histones H3.1 and H3.3 have been identified in some cases of glioblastomas [54]. Moreover, mutations in isocitrate dehydrogenase (IDH1) [55], which regulates the production of a-ketoglutarate required for the enzymatic demethylation of histones and DNA, have been found in ALT tumors (low-grade astrocytoma and multiform glioblastoma). As a result, 2-hydroxyglutarate accumulates, competing with 2-oxoglutarate, and affects DNA/histone methylation, hypoxia signaling, DNA repair, and redox homeostasis, impacting the oncogenesis of IDH-mutated cancers [56,57]. All mutations associated with the ALT mechanism for telomere maintenance promote significant remodeling of telomeric chromatin, making it acceptable for homologous recombination (HR) mechanisms.

Alternative lengthening of telomeres is carried out by proteins that participate in the homology recombination mechanism. Telomeric DNA synthesis in ALT cells involves both intra- and inter-telomeric recombination and replication. ALT cells are characterized by several hallmarks at telomeres: specialized ALT-associated promyelocytic leukemia foci (APBs), heterogeneous telomere length, abundant extrachromosomal telomeric sequences (ECTSs) such as T- and C-circles, and high levels of telomere sister chromatid exchange (T-SCE) [12,58,59]. APBs are unique nuclear structures specific to ALT cells, containing promyelocytic leukemia (PML) protein and telomeric DNA. In APB clusters, telomeres and shelterin associate with recombinase RAD51, RPA [60], DNA resection MRE11-RAD50-NBS1 (MRN) complex [61], Bloom helicase (BLM) [62], and other HR accessory factors such as FANCM, BRCA1 [63], BRCA2, RAD51AP1 [64], and RAD52 [58]. 

In this brief overview, we have described how many proteins are involved in telomere homeostasis; many of them have crucial cellular functions, implying a close relationship between telomeres and cellular state. 

## 3. Non-Telomeric Roles of Telomere Components

### 3.1. Noncanonical Functions of Telomeric Proteins

The telomere is not a self-sustaining structure; rather, it is strongly linked to cellular functions. This relationship is most evident when telomere components execute non-telomeric functions. The regulation of transcriptional control by the telomeric components may be significant in aspects of cellular metabolism regulation. The noncanonical functions of shelterin and telomerase components have recently been reviewed [65]. It is well known that RAP1 is involved in transcription regulation of both subtelomeric and extratelomeric regions. Chip-Seq (chromatin immunoprecipitation sequencing) analysis revealed at least 16 genes involved in cell adhesion, metabolism, and cancer whose expression was affected in RAP1-deficient mouse embryonic fibroblasts (MEFs) [66]. Mammalian RAP1 cannot bind directly to telomeric DNA and requires an interacting partner, TRF2, whose binding to thousands of non-telomeric sites was recently demonstrated. RAP1 regulates the specificity of TRF2 to different genomic regions, influencing transcriptional control [67]. Moreover, TRF2-mediated regulation of VEGF-A (vascular endothelial growth factor A) expression [68] in the process of vascularization and PDGFR-b in the activation of natural killer (NK) cells [69] was demonstrated.

TRF2, in association with nuclear lamina, is involved in organization and stabilization of higher-order DNA looping [70], chromatin compaction, and telomere–interstitial chromatin interaction [71,72]. Recently, it was shown that TRF2 is necessary for the expression of cancer stem cell markers Oct4, Sox2, KLF4, and c-Myc [73].

TRF1 has been shown to be essential for the proliferation of induced pluripotent cells and directly regulated by Oct3/4 [74]. TRF1 involvement in the regulation of cell cycle progression was demonstrated in several investigations. It positively regulates Aurora B kinase during chromosomal segregation [75] and induces cytokinetic failure through direct interaction with the cell cycle regulator Nek2 [76].

### 3.2. Noncanonical Functions of Telomerase Components

The non-telomeric functions of the major components of telomerase have been investigated more intensively. TERT is involved in the regulation of several signaling pathways related to proliferation and metabolism. The activation of the Wnt pathway and proliferation by TERT expression have been demonstrated in mice and human cells. Moreover, the expression of TERT has been shown to be regulated by Wnt. However, the genetic background may affect the results obtained by different scientific groups [77].

It has been demonstrated that TERT regulates inflammation by modulating the expression of NF-kB-dependent genes, suggesting that the interplay between TERT and NF-kB regulates inflammation and development [78,79,80,81].

TERT stabilizes myelocytomatosis oncogene (Myc) protein levels and regulates its binding to target promoters, contributing to either the activation or repression of Myc-target genes, independently of its telomeric role, which influences Myc-dependent oncogenesis [82,83,84]. TERT was shown to regulate the expression of VEGF and tumor development [85,86].

TERT is involved in the long-term regulation of gene expression in cells. It has been demonstrated that TERT expression results in the upregulation of DNMT1 and DNMT3B [87,88]. DNMT3B upregulation promotes de novo methylation of the promoter of the tumor suppressor PTEN, which is involved in the regulation of PI3K/AKT signaling. PTEN inhibition led to an increase in AKT activity, enhanced cell survival, and proliferation [88].

Unlike TERT, the telomerase RNA component is expressed in somatic cells constitutively, and its alternative non-telomerase functions have been demonstrated. TR (telomerase RNA component) is involved in the regulation of gene expression by direct complementary interaction with promoter regions of Wnt, Myc target genes, along with several genes related to NF-kB-pathway and genes involved in developmental myelopoiesis [89,90,91,92].

Telomerase components are also involved in cell protection mechanisms. Both TR and TERT are involved in DNA damage response. It has been demonstrated that TERT influences the phosphorylation of yH2AX-histone and ATM (ataxia-telangiectasia mutated) autophosphorylation [93], while TR is involved in the regulation of ATR kinase activity [94]. TR overexpression promotes T-cell survival under apoptosis induction conditions [95].

### 3.3. Telomeric and Telomerase Components and Mitochondria

The interconnection between telomeres and mitochondria plays a role in genome stability and cell fate. The mitochondrial electron transport chain (ETC) provides the transmembrane proton gradient used by ATP synthetase to produce ATP during oxidative phosphorylation (OXPHOS), sufficient for the energy supply of cell needs [96]. Controlled leakage of electrons carried by the respiratory chain to oxygen results in the production of reactive oxygen species (ROS). At low levels, these ROS work as signal molecules, but at high levels, they have the potential to damage all types of biomolecules [97]. Eukaryotic cells have antioxidant mechanisms to protect against oxidative stress by balancing ROS production and utilization through enzymatic and non-enzymatic processes. However, an imbalance between ROS production and cellular defense mechanisms results in oxidative stress, which is believed to be highly damaging to telomeric DNA because it is enriched with guanine residues, which are more sensitive to oxidation, producing 8-oxo-7,8-dihydroguanine (8-oxoG). Moreover, DNA damage repair is repressed at telomeres. The existence of a correlation between oxidative stress and telomere attrition in short-lived species has been demonstrated in vitro and in vivo under physiological conditions [98,99,100,101]. On the other hand, recent data also support the view that mitochondrial ROS are not directly involved in DNA damage overall, or at telomeres in particular, although the occurrence of damage to nuclear DNA during oxidative stress of mitochondrial origin is undisputed [102].

One of the shelterin components, TIN2, contains an N-terminal mitochondrial localization signal, which overlaps with the TPP1-interacting domain and normally targets TIN2 to the telomeres. Thus, the localization of TIN2 is regulated through the abundance of TPP1. TIN2 is processed to the shorter form through proteolytic cleavage in mitochondria. It is involved in the regulation of glucose metabolism and production of ATP [103].

Surprisingly, the major components of telomerase, TERT and TR, are localized in mitochondria and regulate the functioning of this organelle. Human TERT contains an N-terminal leader sequence, which is used for targeting in mitochondria upon oxidative stress in various human cells [104,105]. The overexpression of TERT increases the mitochondrial membrane potential and reduces the superoxide level via an unknown mechanism [106,107,108]. It has been proposed that hTERT regulates the expression of SOD2 superoxide dismutase and proteins involved in ATP synthesis [108,109,110,111,112,113]. Thus, hTERT can not only regulate utilization but can also indirectly control the production of ROS, which positively correlates with the value of the membrane potential spent during the work of F_0_F_1_ ATP-synthase. It is known that, under physiological conditions, a decrease in membrane potential during ATP synthesis in intact, isolated mitochondria or cells leads to a decrease in ROS levels, reflecting complex reciprocal relationships between metabolic activity, ROS-mediated signaling, and oxidative stress in living cells [114,115,116]. Interestingly, the paradox of the steep H_2_O_2_ gradient around the mitochondria, which is often cited as the main argument against mitochondrial ROS damaging nuclear DNA [116], along with the shuttle transfer of TR, TERT, and microRNA between the nucleus and mitochondria (for review, see [117]), can be resolved by considering the localization of mitochondria directly within the nucleus. While TR has also been observed in mitochondria, it is processed into a shorter form before being re-exported to the cytoplasm, enabling the sensing of mitochondrial membrane potential [118,119]. First proposed in 1958 and long rejected, this concept has recently found experimental confirmation and helps explain a number of paradoxes of mitochondrial–nuclear cross-talk; however, further studies are needed to elucidate the significance and mechanisms of such interactions in higher eucaryotes, as well as the mechanisms by which telomerase reduces ROS, which remain underexplored and require further investigation [120,121].

Interestingly, telomerase is also involved in mitophagy and mitochondrial biogenesis activation for the recycling of damaged mitochondria [122]. Recently, it was shown that mitochondrial protein methylcrotonoyl-CoA carboxylase, responsible for the enzymatic conversion of 3-methylcrotonyl-CoA to 3-methylglutaconyl-CoA in the catabolism of aliphatic amino acids, is responsible for telomerase–mitochondria interaction through its competitive binding and complex formation with the telomere binding protein TRF2; this complex is suggested to be critical for telomerase recruitment to telomeres [123]. In knockout cell models, both the number of mitochondria (through the upregulation of fusion markers MFN1, MFN2 and OPA1) and telomere lengths were reduced in the absence of methylcrotonoyl-CoA carboxylase.

Furthermore, since TR encodes the regulatory protein hTERP, it may control the autophagy program [124]. Anabolic growth of cells is regulated by genes activated by the mammalian target of rapamycin (mTOR), a serine/threonine kinase that integrates multiple extra- and intracellular signals and promotes glycolysis, growth, and proliferation [125,126]. mTOR is regulated by a sensor of the AMP/ATP ratio, AMPK, which drives catabolic metabolism when energy stores are depleted. It stimulates mitochondria biogenesis and inhibits mTOR activity. Both mTOR and AMPK form an axis of reciprocal regulation of catabolic and anabolic pathways [127,128]. Interestingly, the hTERP protein encoded in the precursor of the human telomerase RNA component is involved in the regulation of autophagy and cell proliferation through the AMPK pathway and stimulates autophagy and proliferation [124,129]. Autophagy is activated when glycolysis is inhibited, so we could hypothesize that the switching between the biogenesis pathway of the primary transcript of the telomerase RNA gene [130] may be regulated by intracellular signals and metabolic reprogramming to modulate the telomerase activity in accordance with cell’s requirements for proliferation rate.

## 4. Metabolic Programs and Telomere Elongation

Telomere maintenance mechanisms are activated during certain physiological stages and processes that require an increase in the cell proliferation rates [131,132]. Proliferation rate activation requires increased levels of nutrients for the synthesis of components necessary for building new cells. Moreover, it is becoming clear that metabolic pathways can also play modulatory or instructive roles in the regulation of cellular programs, which can be summarized as metabolic signaling functions [133,134].

**Figure 4:**
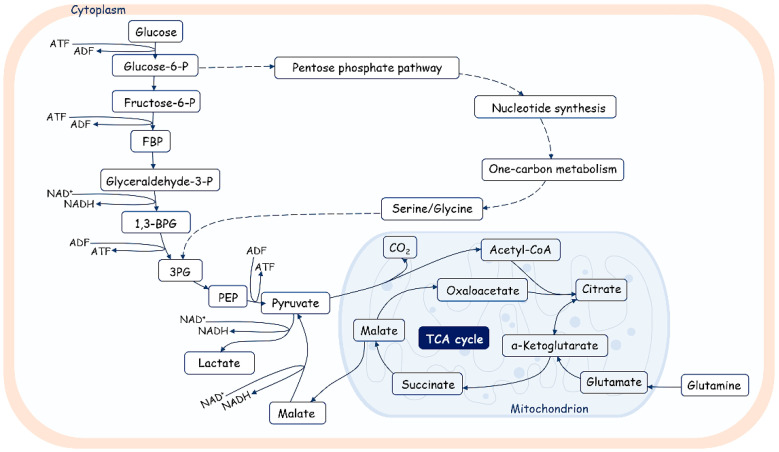
An overview of glucose metabolism. Glucose is catabolized in a series of enzymatic reactions yielding pyruvate. Pyruvate is converted in lactate or transported in mitochondria and metabolized by pyruvate dehydrogenase (PDH) into acetyl-CoA, fueling the tricarboxylic acid cycle (TCA). FBP—fructose biphosphate; 1,3-BPG—1,3-biphosphoglycerate; 3PG—3-phosphoglycerate; PEP—phosphoenolpyruvate. Glycolysis also fuels the pentose phosphate pathway, which produces nucleotides, amino acids, and fatty acids.

The majority of cells in normal physiological status use oxidative phosphorylation (OXPHOS) as the main metabolic program. However, in cases of increased proliferation rates and oxygen deficiency in lesions or neoplasia, OXPHOS switches to the glycolysis program in satellite stroma cells (cancer-associated fibroblasts in the tumor microenvironment, for example), whereas in proliferating cells, OXPHOS increases. The formation of the so-called metabolic symbiosis between glycolytic stroma cells and proliferating cells provides a chance to generate enough energy to fulfill the requirements of dividing cells through the import of lactate and pyruvate from the intercellular space (Figure 4) [133,134,135]; for review, see also [136]. It is of note that intermediate metabolites affect downstream biochemical reactions and protein modifications, such as protein acetylation, glycosylation, and methylation [137].

It is well known that metabolism is regulated over time and space at the intercellular, subcellular, and tissue levels. Metabolism is regulated during cell cycle progression. Glycolysis is activated during the G1/S transition, while mitochondrial respiration increases during the G2/M transition [138,139,140,141]. The reduced OXPHOS activity at the stage of DNA replication should minimize the risk of oxidative damage to DNA due to ROS produced by mitochondria in stroma cells. Moreover, acetyl-CoA—the main substrate for energy production by mitochondria—originates in proliferating cells mainly from imported lactate. It is also used for the acetylation of histones, promoting the epigenetic regulation of gene expression, and its levels should be enhanced during DNA synthesis and the establishment of epigenetic marks [142]. The epigenetic state of chromatin is also regulated by the level of other metabolic intermediates. The level of a-ketoglutarate is important for maintaining the methylation of DNA and histones, thereby regulating the expression activity of the genome [143].

It is interesting that during the cellular phase in which telomerase elongates telomeres, the glycolytic metabolism program is activated; moreover, in cells with an increased proliferation rates where telomeres need to be elongated, mitochondrial OXPHOS activity is enhanced, and glycolysis continues to provide the necessary resources for the synthesis of compounds for new cells.

### 4.1. Cellular Metabolic Programs and Telomere Elongation in Cancer Cells

The link between metabolism and telomere maintenance is mostly addressed in cancer cells. Cancer cells activate glycolysis in cancer-associated fibroblasts (CAFs) to import the nutrients needed for making new cells. In transformation and cancer progression, the cellular environment changes. For example, it has been demonstrated that during the first steps of tumor growth, cells are in a state of hypoxia, but further development promotes vascular growth, and the level of oxygenation reverses to a normal range. Moreover, tumor cells activate glycolysis via a mechanism known as the Warburg effect, but the OXPHOS mechanism is hyperactivated in cancer cells during cancer progression [144]. Cancer cells proliferate faster because of changes in certain mechanisms that lengthen telomeres. These changes can be caused by mutations in promoter regions [145,146,147], resulting in the expression of hTERT and activation of telomerase. Some metabolites provide the resources for chromatin modifications, which influence the regulation of expression activity. Acetyl-CoA provides the acetyl group for histone acetylation, while a-ketoglutarate is used for maintaining the methylation of histones and DNA. Changes in the metabolic program during oncogenic transformation of cells may affect long-range chromatin organization in the promoter region of the hTERT gene [148], followed by the mutation of this region, leading to the activation of expression. The hTERT supports noncanonical functions and regulates the expression of genes that are involved in cancer cell metabolism [149]. Furthermore, telomere proteins control many aspects of cellular functioning, especially in cancer cells [150,151], and may influence metabolism, inflammation, and repair. The decrease in these repair processes leads to the activation of a mechanism called alternative lengthening of telomeres, which is based on a type of DNA recombination [19].

Next, we will focus on the mechanisms of switching the metabolism program and telomere lengthening mechanisms during development and T-cell activation. In contrast to cancer cells, in normal development, the activation of telomere lengthening is transient and switched off after a short period of telomere elongation coupled with increased proliferation.

### 4.2. Metabolism and Telomere Lengthening during Gametogenesis and in Early Development

Mammalian organisms start to develop from fertilization, where two specialized cells, the spermatozoa and the oocyte, fuse together to form a totipotent 1-cell zygote. Further division, coupled with the differentiation of newly obtained cells, gives rise to the whole embryo and extra-embryonic tissues, such as trophoblasts, which give rise to the placenta at later stages of development. Successful growth of a new organism requires proper reorganization of chromatin that is restricted in time and coordinated with the stage of development. Proper telomere length is crucial for organizing the genome of eukaryotic organisms. It enables cells to divide multiple times during development, because both mechanisms of telomere lengthening are used at different stages.

Gametogenesis represents a pivotal phase preceding embryonic development, wherein germline cells, having colonized the gonadal tissue, engage in successive processes of active proliferation and meiotic division, leading to substantial cellular specialization. In the field of telomere biology, a prevailing consensus asserts that germ cells prevent the attrition of telomeres through the active expression of telomerase, and then, as embryonic development ensues, telomeres undergo gradual reduction with each cycle of DNA replication [20]. Nonetheless, there exists a significant disparity in telomerase activity between oogenesis and spermatogenesis.

#### 4.2.1. Metabolism and Telomere Lengthening during Spermatogenesis

**Figure 5:**
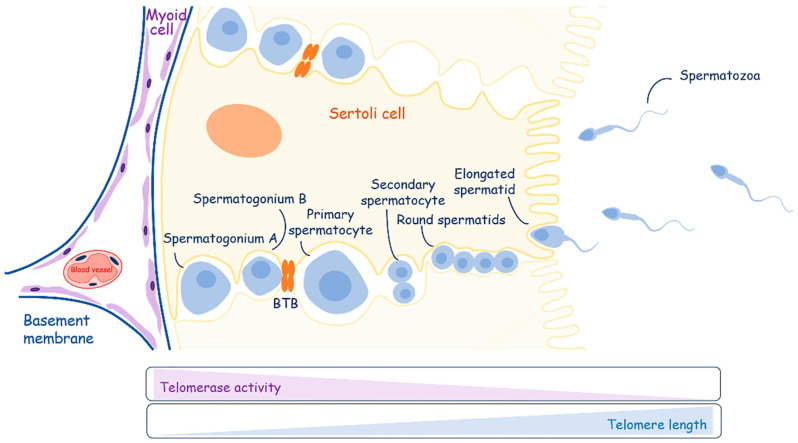
A schematic overview of spermatogenesis. Sertoli cells play an important role in energy and nutritional support of developing germ cells. Telomerase activity decreases during the differentiation of spermatogonia to mature spermatozoa, contributing to the elongation of telomeres.

Sperm cells undergo maturation, which is accompanied by telomere elongation and a decrease in telomerase activity [152] (Figure 5). However, the data on telomerase activity in different types of male germ cells during differentiation are controversial, which may be attributed to the difficulties in obtaining pure populations of cells from specific stages of differentiation. It was observed that late generations of mice lacking telomerase RNA (mTR−/−) are sterile. In these animals, male germ cells are depleted, showing that telomerase activity is required for effective spermatogenesis. The majority of research findings have consistently indicated that men experiencing idiopathic infertility tend to exhibit shorter telomere lengths in comparison to their fertile counterparts [153]. Telomere length is positively correlated with sperm count, motility and the ratio of high-quality and transferable embryos [154]. Consequently, contemporary research places considerable emphasis on telomere length as a highly promising marker for evaluating male reproductive biology [155].

The maturation process of spermatogonial stem cells occurs in the testicles, which also produce sex steroid hormones. They consist of the seminiferous tubules and the intervening interstitial space. Sertoli cells create the blood–testis barrier through junctions between adjacent cells and compartmentalize the seminiferous epithelium. The blood–testis barrier consists of three components: a physical barrier that restricts the movement of molecules and stem cells from the basal compartment to the abluminal compartment of seminiferous tubules; an immunological barrier that regulates the movement of immune cells and the levels of cytokines; and a physiological barrier composed of transporters and channels that supply necessary compounds to germ and Sertoli cells [156]. 

Sertoli cells work as “nurses” (CAF analogs) and are responsible for providing energy in the form of pyruvate or lactate and nutritional support to developing germ cells. Mammalian spermatogenesis is a process of cellular differentiation with three main stages: mitotic spermatogonial proliferation and differentiation; meiotic phase; and spermatogenesis. Spermatogonial cells, which lie at the basement membrane, replicate mitotically to support the population of germ stem cells (spermatogonia A) and give rise to new populations (spermatogonia B) committed to differentiate and move along the seminiferous epithelium. Spermatogonia B differentiate into primary spermatocytes, cross the blood–testis barrier, and undergo the first division of meiosis, yielding secondary spermatocytes. Round spermatids are produced through the second meiotic division. After meiosis, cell division stops, and spermatogenesis starts, giving rise to elongated spermatids. Elongated spermatids are released into the lumen of the tubule as immature spermatozoa. During differentiation, germ cells have specific metabolic requirements, switching their metabolic profile throughout development. They are supported with lactate [157], which is produced by Sertoli cells via the metabolism of various substrates, preferentially glucose [158]. 

Thus, both aerobic and anaerobic pathways of carbohydrate metabolism are vital for germ cells [159,160]. Spermatogonia are supplied by blood components and use glucose for ATP production via the aerobic pathway. Spermatocytes—intermediate developing germ cells—combine two metabolic pathways and also depend on glycolysis. The glycolytic activity of spermatids is lower than in germ cells at earlier developmental stages, and the ATP level in spermatids decreases. However, round spermatids support both glycolytic and gluconeogenesis pathways in order to recycle lactate to glucose-6-phosphate. Spermatozoa exhibit the highest glycolytic activity and the lowest tricarboxylic acid (TCA) cycle activity, using only glucose or fructose for their energy metabolism. Moreover, different pathways are used in different compartments of spermatozoa: OXPHOS is restricted to the midpiece, while glycolysis is used in the principal piece [161,162].

Taken together, during development, spermatozoa switch from the metabolic program of OXPHOS in spermatogonium to glycolysis in round spermatids. Further differentiation is supported through the compartmentalization of metabolic pathways for site-specific requirements in metabolites. Moreover, DNA replication does not occur during the metamorphose of round spermatids to the spermatozoa, so high telomerase activity should not be necessary. 

It is important to note that the testes are naturally oxygen-deprived organs, making male germ cells susceptible to oxidative stress, which is negatively associated with sperm DNA fragmentation [163,164]. Abundant evidence underscores the significant role of reactive oxygen species (ROS)-mediated sperm damage as a primary contributing factor to infertility among patients [165]. Sperm, due to their relatively limited antioxidant defense mechanisms and their specific mitochondria structure, are particularly vulnerable to elevated oxidative stress levels. Oxidative stress can be risky for sperm DNA and RNA, and can also affect the integrity of telomeres and telomerase activity. This can lead to shorter telomeres [166].

#### 4.2.2. Metabolism and Telomere Lengthening during Oogenesis

**Figure 6:**
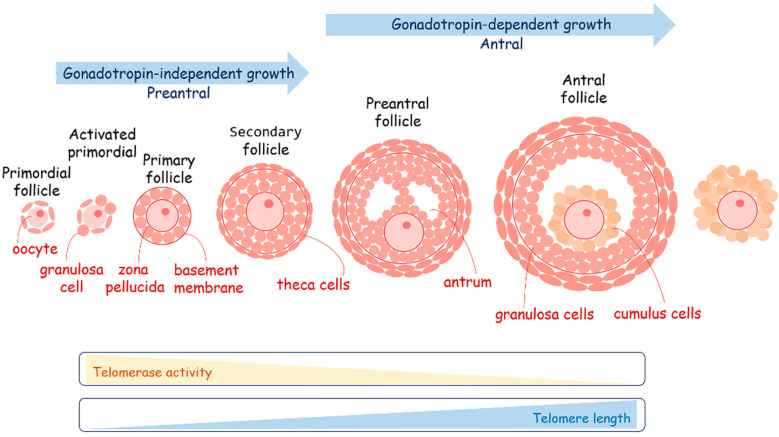
A schematic overview of oogenesis. The maturation of oocytes is very sensitive to metabolism coordinated by the follicular cells. Telomerase activity decreases during oogenesis, providing the elongation of telomeres.

In contrast to the general trend of “greater potency equating longer telomeres,” the female germline in many mammalian species stands as an exception. Notably, telomeres within mouse and human oocytes are among the shortest observed throughout the body, and they exhibit a low to absent level of telomerase activity [20,167]. The divergence in telomerase activity between oogenesis and spermatogenesis manifests to varying degrees across distinct mammalian species (Figure 6). A noteworthy illustration can be found in the Tasmanian devil and other marsupials, where an exceptional degree of telomere length dimorphism in gametes has been elucidated. This phenomenon involves a distinctive pattern: all telomeres in spermatozoa undergo hyperelongation, whereas those in oocytes experience hypershortening [168].

Nonetheless, in the case of mice, humans, and many other mammals, oocyte telomere length is delicately balanced and serves as an indicator of their quality. Telomerase activity measurements conducted through in vitro TRAP (telomerase repeated amplification protocol) assays have unveiled a distinctive pattern: a peak in pre-ovulation oocytes followed by a subsequent decline in mature oocytes [169,170]. The telomere length is directly associated with the developmental potential of oocytes. Oocytes with shortened telomeres resulting from telomerase-null mice exhibit a striking pattern of failure, occurring during both fertilization and the early cleavage embryonic stages [171]. This observation supports the hypothesis that low telomerase activity may serve as a selective mechanism, favoring the successful fertilization of egg cells that have already attained adequate telomere length during oogenesis [172]. This is predominantly because telomere attrition substantially promotes genomic instability through mechanisms like non-homologous end joining, ultimately resulting in conditions such as aneuploidy, mosaicism, and the emergence of chromosome copy number variants [173]. The shortening of telomeres in telomerase-null mice has been linked to several detrimental outcomes in oocyte development. These outcomes include the formation of abnormal meiotic spindles [174], the arrest and fragmentation of embryos [175], a decrease in chiasmata and synapsis [176], and ultimately, infertility. Furthermore, oocyte telomere length has been associated with various challenges in in vitro fertilization (IVF) cycles, such as failed IVF cycles, embryo fragmentation [175], and blastocyst aneuploidy [177]. These findings underscore the critical role of telomere length in oocyte health and reproductive success. The diminishing ovarian reserve that occurs in tandem with advancing female age leads to a concomitant telomere depletion, which holds a central role in the process of oocyte aging. This is supported by the discovery of telomere shortening in oocytes obtained from females of advanced reproductive age [178]. Age-related telomere shortening is primarily attributed to the enduring negative consequences of reduced telomerase activity and heightened exposure to reactive oxygen species (ROS) [178,179]. Research findings have demonstrated that the application of antioxidants enhances the quality of oocytes obtained from older females, including an increase in both telomerase activity and telomere length [180].

The process of oogenesis occurs in close collaboration with somatic follicular cells, specifically granulosa cells (Figure 6). The maturation of oocytes is very sensitive to metabolism, coordinated by the follicular cells. The oocytes preferentially use the pentose phosphate pathway to metabolize glucose instead of glycolysis. They obtain pyruvate as a fuel for the TCA from follicular cells with which they are also in a state of metabolic symbiosis. It is curious that during in vitro cultivation of oocytes obtained through the IVF program and early embryos after fertilization, pyruvate is an essential component of the growth medium. During the period of follicular growth, granulosa cells exhibit robust proliferative activity and elevated levels of telomerase activity [181,182,183,184]. The length of telomeres in granulosa cells plays a pivotal role in regulating the normal progression of folliculogenesis and overall ovarian function. Reducing telomerase activity in granulosa cells has been associated with an elevated rate of apoptosis and an increase in the number of atretic (degenerating) follicles [182,184]. In granulosa cells, the activity of telomerase and the length of telomeres are regulated by estrogen levels [185,186]. High concentrations of estradiol-17β have been shown to significantly increase the telomere length of granulosa cells cultured in vitro [187]. Conversely, the withdrawal of estrogen consistently results in reduced telomerase activity, which may lead to telomere shortening in granulosa cells, subsequently contributing to follicular atresia [184,188]. Interestingly, estrogen was shown to play a significant role in the regulation of metabolism. It acts as a transcriptional factor regulating the expression activity of metabolic genes involved in OXPHOS and glycolysis switching or directly influencing mitochondrial activity because of estrogen receptor localization on the mitochondria membrane close to the cholesterol transport system [189,190]. Mitochondria are the central sites for steroid hormone biosynthesis. The first and rate-limiting step in the biosynthesis of steroid hormones occurs in the mitochondria of granulosa cells [191]. The localization of the estrogen receptor in close proximity to cholesterol transport opens intriguing possibilities for the regulation of estradiol production. Short telomere length and the absence of telomerase activity in the granulosa cells of women have been associated with occult ovarian insufficiency [192]. Indeed, short telomere length has been reported in young patients who exhibit a low ovarian response to hormonal stimulation [193]. This observation highlights the potential significance of telomere length as a predictor or indicator of ovarian responsiveness and reproductive health in individuals of reproductive age. Cumulus cells demonstrate high glycolytic activity for the production of pyruvate, which is transported to the oocytes through gap junctions or is directly taken up from follicle fluid. During ovulation, a subset of the follicular cells that envelop the oocyte accompanies it as part of the cumulus–oocyte complex [194]. It was observed that follicle-stimulating hormone (FSH)-dependent mitochondrial elongation shortly after stimulation led to mitochondrial fragmentation on the next day with a subsequent decrease in mitochondrial activity and a switch to glycolysis in cumulus cells [195]. These cumulus cells can be conveniently obtained alongside oocytes, presenting significant diagnostic potential when employing assisted reproductive technologies. The relative telomere length tends to be greater in cumulus cells derived from good-quality embryos as compared to cumulus cells associated with embryos of lesser quality [196]. This finding highlights the utility of assessing telomere length in cumulus cells as an effective means of evaluating embryo quality in the context of assisted reproduction and that the metabolic environment plays a crucial role in germ cell maturation and telomere maintenance [197].

#### 4.2.3. Metabolism and Telomere Lengthening during Early Development

**Figure 7:**
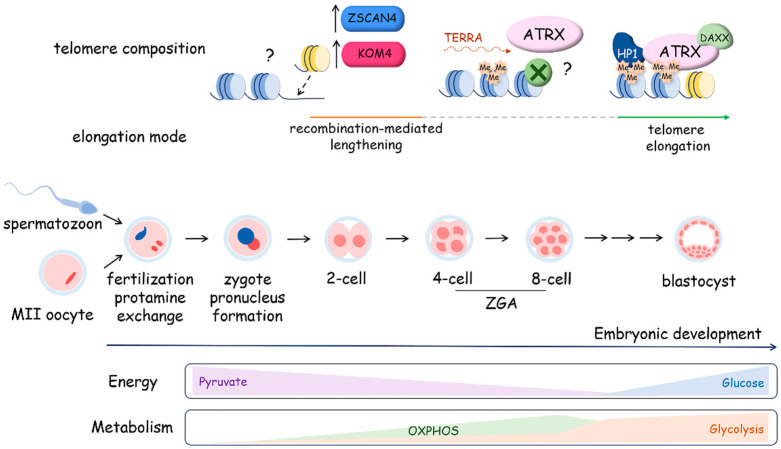
Telomere dynamics and metabolic program preferences during mouse embryo preimplantation development. During the first stage following fertilization, extensive chromatin remodeling occurs with histone-to-protamine replacement, the formation of two parental pronuclei and major zygotic genome activation (ZGA) at the two-cell stage, accompanied by the elongation of telomeres via a recombination-mediated mechanism that is telomerase-independent and involves the activation of the OXPHOS metabolism program. Then, at the stage following the morula–blastocyst transition, telomerase is activated. This process is characterized by a switch in the metabolism program from OXPHOS to glycolysis. In mouse zygotes, at the morula stage, ATRX is targeted to telomeres. The expression of H3K9 lysine demethylases from the *Kdm4* family and *Zscan4* favors telomere elongation through recombination in mouse and human zygotes.

The early stages of mammalian development involve the dynamic regulation of telomere length in embryonic cells, as observed in various mammalian species, including mice, rats, cows, and humans (Figure 7). Telomere length tends to increase during preimplantation development, reaching its peak at the blastocyst stage [198]. Remarkably, this elongation process occurs even in the absence of telomerase activity during the early cleavage stages, spanning from two to four cells up to the morula stage. Telomeres undergo elongation through an ALT-like mechanism during these early developmental stages. Recombination-mediated telomere lengthening at the early cleavage stage is driven by telomeric chromatin reorganization due to H3K9 demethylation by KDM4 and Zscan4. The maintenance of Zscan4 activity in early embryos and 2-cell-like embryonic stem cells is facilitated by Dcaf11 (Ddb1- and Cul4-associated factor 11) [199,200]. Moreover, the transcription of TERRA is activated during early cleavage stages, confirming the open conformation of telomeric chromatin. TERRA accumulation at telomeres promotes ATRX recruitment followed by the attraction of HP1 and compactization of the telomeres at the morula stage, accompanied by the inhibition of ALT-like mechanism telomere elongation and activation of telomerase, providing telomere lengthening at the blastocyst stage [201].

Interestingly, zygotic gene activation, which occurs at the early cleavage stage, is supported by the regulation of the metabolic program. During the early cleavage stage of development, the mitochondrial enzymes responsible for the production of acetyl-CoA and a-KG are transiently localized to the nucleus, where they impact epigenetic histone acetylation, promoting chromatin opening [202]. It was demonstrated that mitochondrial pyruvate dehydrogenase (PDH) complex is phosphorylated and inhibited in cleavage-stage embryos, but nuclear PDH remains unphosphorylated and active, which influences the epigenetic regulation of genome expression. Pyruvate metabolism supports the development of the embryo upon fertilization, and glucose catabolism becomes activated at the eight-cell stage and is associated with the inhibition of ALT-like telomere lengthening and activation of telomerase, which elongates telomeres up to the blastocyst stage and is inactivated during cell differentiation.

At the blastocyst stage, the maximum telomere length and mitochondria number are observed, exceeding those observed both at earlier stages of preimplantation development and during subsequent embryogenesis in correlation with an increase in oxygen consumption [203,204]. Telomerase activity emerges as a crucial factor driving telomere elongation during the transition from morula to blastocyst. Analysis of telomere length in eight-cell, morula and blastocyst-stage embryos obtained from mTR−/− or mTR+/+ mouse models revealed that blastocysts from mTR+/+ mice displayed significantly longer telomeres compared to those at the eight-cell and morula stages. Conversely, blastocysts from mTR−/− mice did not exhibit this telomere elongation [198]. The blastocyst comprises two main cell types: the trophectoderm (TE) covering the outer surface of the embryo and the inner cell mass (ICM) situated inside of the blastocyst. While the ICM forms embryonic structures, the TE has more limited potential and contributes to the development of extraembryonic membranes. Despite these differences in cell fate, telomere length shows only minor variations between the cells of the inner cell mass and the trophoblast, making the latter a convenient object for measuring telomere length during preimplantation development [198]. Telomere elongation during the blastocyst stage significantly influences telomere length during subsequent stages of development. In comparing telomerase activity in bovine embryos undergoing development in vivo, in vitro, and after in vitro fertilization (IVF) and parthenogenetic activation, researchers noted only minor quantitative differences [198]. However, a recent study involving children born through assisted reproductive technology (ART) revealed that those born following a blastocyst transplant procedure exhibited shorter telomeres in their white blood cells by the age of one year compared to their naturally conceived counterparts [205]. Furthermore, research conducted in mice has shown that in vitro culture of mouse embryos suppresses telomerase activity during the early blastocyst stage, which subsequently leads to telomere shortening. The intricate and dynamic regulation of telomere length during early development renders this mechanism susceptible to various negative influences, both external and internal. For instance, studies have demonstrated that a high-fat diet and obesity in female mice can lead to reduced telomerase activity and telomere shortening in oocytes and early embryos, indicating the importance of cellular metabolism in telomere maintenance [206].

Embryonic stem cells (ESCs) obtained from the ICM of a blastocyst have longer telomeres than mouse embryonic fibroblasts (MEFs) from the same genetic background, which are typically obtained at embryonic day 13.5. This confirms that telomeres shorten after the blastocyst stage during cell differentiation to promote embryo development. The generation of induced pluripotent cells (iPSCs) from MEFs is accompanied by telomere elongation up to a length similar to the telomeres in ESCs. Moreover, telomeres are elongated in MEFs during cultivation, and at early passages (P5), their length is comparable with that of the telomeres in the ICM of the blastocyst. It was demonstrated that decreased levels of heterochromatin mark H3K9me3 and H4K20me3 at subtelomeric regions of ESCs and in iPSCs that could activate the mechanisms of telomere lengthening [203].

Interestingly, a correlation between the metabolism program, telomere length, and ability to differentiate was recently demonstrated for human embryonic stem cells (hESCs). It was determined that the expression level of genes involved in the OXPHOS metabolic pathway is increased in hESCs with short telomeres; however, the expression level of genes related to glycolysis metabolism is upregulated in cells with long telomeres and active telomerase [207]. Moreover, pluripotent ESCs are characterized by high glycolysis activity, but primed and differentiated cells demonstrate increased OXPHOS activity [208].

Taken together, the experimental data allow the proposal that telomere lengthening in the processes of gametogenesis, early development, and differentiation of stem cells is tightly dependent on the chromatin status of telomeric and subtelomeric regions, which is regulated by the accessibility of components for epigenetic modifications obtained from metabolic processes and mitochondrial status in the cell (for review, see [209]).

### 4.3. Metabolism and Telomere Lengthening during Immune Cell Activation

The switching between different metabolic programs in T-cells during their development and maintenance is well studied. Quiescent T-cells use catabolic pathways such as OXPHOS, which provide efficient and robust energy output, while the immune activation of T-cells is supported by an anabolic glycolysis pathway, which provides nutrients necessary for protein production and cell division. The activation of T-cells requires increased proliferation, which is accompanied by telomerase activation and telomere lengthening [132,210]. Naïve and memory T-cells maintain their energy levels by relying on OXPHOS, as their energy demand is relatively low during homeostasis in a quiescent state. Upon activation, T-cells proliferate at an incredibly fast rate and differentiate into effector cells. Early upregulation of glycolysis during T cell activation is supported by the activation of PDH kinase 1, which phosphorylates and inactivates PDH, leading to the inhibition of OXPHOS and the engagement of aerobic glycolysis [134,211]. T-lineage progenitor cells undergo differentiation and selection in the thymus. Mature T-cells leave the thymus and reside in the peripheral lymphoid tissues while circulating throughout the lymph nodes and blood. It has been well documented that telomerase activity is tightly regulated during T-cell lineage development. The highest level of telomerase activity was detected in progenitor cells and in thymocytes from the thymus, where cells use glycolysis to obtain the necessary nutrients for proliferation. Telomerase activity decreased in resting memory T-cells when cells do not proliferate and reactivated in memory cells under stimulation conditions simultaneously with the activation of proliferation supported by glycolysis. However, the long-term stimulation of memory T-cells results in decreased telomerase activity and telomere shortening. It was observed that culture conditions unsuitable for the proliferation activation do not stimulate telomerase activity in T-cells [211,212].

Unfortunately, direct analysis of the association between telomerase activation and metabolism switching during immune cell activation has not been conducted to date, and we can only highlight certain events that occur at the same stage of the activation process. 

## 5. Targets and Approaches for Telomere Reprogramming

Telomere reprogramming is an attractive target for the development of approaches and drugs that influence telomere length. Long telomeres enhance the lifespan of healthy cells; however, the elongation of telomeres is associated with cancer cell survival and propagation. The telomerase holoenzyme is considered a promising target for the regulation of telomere length. Inhibitors of telomerase activity are being developed as potential anticancer drugs; however, activators of telomerase activity may be considered as a means to increase the lifetime of the organism. Recently, the oligonucleotide derivative GRN163L was approved by the U.S. Food and Drug Administration (FDA) to treat low- to intermediate-risk myelodysplastic syndromes in adults who do not respond to erythropoiesis-stimulating agents. GRN163L is a lipidated 13-mer thiophosphoramidate oligonucleotide that, by directly hybridizing to hTR with very high affinity and specificity, is able to competitively inhibit telomerase activity [213,214]. A lot of different substances regulate the expression level of hTERT at the transcriptional level by influencing the promoter region of the gene. One of them, TA-65, was demonstrated to be a positive regulator of telomere length; however, it has not been thoroughly investigated to exclude potential side effects such as stimulating cancer [215].

The treatment with FDA-approved 5-fluoro-2′-deoxyuridine (5-FdU) triphosphate results in misincorporation in telomeres followed by rapidly inducing telomere dysfunction and cell death in telomerase-expressing cells. Other nucleotide derivatives, such as 6-thio-2′-deoxyguanosine, demonstrate similar effects on telomere function [216,217].

Despite the absence of approved drugs at this time, G-quadruplex ligands are considered a promising target for telomere reprogramming. The stabilization of G-quadruplexes should prevent telomerase binding and elongation of the G-overhang of telomeres, displace shelterin components and stabilize DNA loops in the telomeric region during replication or R-loop formation, leading to replication- and transcriptional-dependent damage [218,219,220].

The shelterin components may be targeted to induce telomere reprogramming. TRF1, TRF2 and POT1 proteins are targets for post-translational modifications that regulate their telomeric binding properties. Therefore, some kinase inhibitors used in cancer treatment approaches reduce the recruitment of shelterin components to telomeres.

Interestingly, in addition to approaches that manipulate telomeres by directly influencing telomeric DNA structure or shelterin proteins, some medications for the treatment of metabolic disorders demonstrate the ability to prevent telomere shortening. For example, increased telomere shortening is associated with diabetes mellitus [221], and medical care with metformin slows down the attrition of telomeres [222]. Metformin is a therapeutic substance that normalizes insulin sensitivity in cells and regulates metabolic processes at the organismal level [223]. The telomere-protecting effects of other exogenous factors are also discussed. For example, it has been demonstrated that physical activity, healthy diet, and other factors that improve metabolism decrease the rate of telomere shortening [224]. Approaches directed at metabolic reprogramming should be recommended as potential regulators of telomere length at the organismal level, influencing the functions of immune, reproduction, and other systems in the organism.

## 6. Conclusions

Cell proliferation is important for organism development, regeneration, immune system function, cancer progression, and more. Cell division relies on energy, nutrition, metabolism regulation, genome integrity, and the maintenance of telomere length and structure. In this review, we analyzed the mechanisms of metabolism and telomere maintenance in processes of proliferation activation. We focused on different models that have experimental data on homogeneous cellular populations. These models have well-studied metabolism reprogramming and information on telomere elongation. This review provides evidence that specific metabolic conditions within proliferating cells are associated with telomere reprogramming and are likely directly involved in the regulation of telomere lengthening mechanisms. The study of the molecular mechanisms underlying the relationships between telomeres and metabolic pathways appears to be promising for identifying strategies to influence telomere maintenance. The ability to use specific metabolites to regulate telomere length and cell proliferative activity may be important in the development of approaches to regeneration, immune response modulation, cancer therapy, and age-related conditions. The most promising application may be in immunotherapy, where stimulating telomere lengthening through metabolic manipulation could provide the necessary proliferation rate for exogenous immune cells. Another attractive area of application is stimulating telomere lengthening during in vitro fertilization and early embryo development to produce a blastocyst with proper telomere length, potentially ensuring a normal or improved lifespan for the organism. Investigating the metabolic status of cells, telomerase activity, and telomere length in each cellular population under specific conditions and during changes in the proliferation activity is crucial for understanding the biology of aging. This knowledge can also inform natural approaches for prolonging healthy lifespan, as well as providing insights into cancer progression and treatment.
